# High-Throughput Qualitative and Quantitative Drug Checking by MALDI HRMS

**DOI:** 10.3389/fchem.2020.00695

**Published:** 2020-08-25

**Authors:** Timothée Joye, Christèle Widmer, Roxane Morger Mégevand, Serge Longère, Marc Augsburger, Aurélien Thomas

**Affiliations:** ^1^Forensic Toxicology and Chemistry Unit, CURML, Lausanne University Hospital, Geneva University Hospitals, Geneva, Switzerland; ^2^Faculty Unit of Toxicology, CURML, Faculty of Biology and Medicine, University of Lausanne, Lausanne, Switzerland; ^3^Nuit Blanche?, Association Première Ligne, Geneva, Switzerland

**Keywords:** MALDI, drug checking, qualitative, quantitative, HRMS, high throughput

## Abstract

Illicit drugs are a global health problem, since both their acute and chronic consumption have negative impacts on the drug user's health. Drug checking facilities are receiving growing interest as they allow drug users to chemically analyze their product prior to consumption to assess the presence of adulterants or other non-expected substances. Such harm reduction programs allow the reduction of the risks associated with drug consumption without encouraging it. In particular, the emergence of new psychoactive substances (NPS) emphasizes the risk for the population increasing the diversity and the lability of illicit drugs on the market. Analytical developments are required to catch up with this rapid evolution and reduce the potential harm caused by such consumption. In this study, we developed a matrix-assisted laser desorption/ionization (MALDI) high-resolution mass spectrometry (HRMS) strategy for the high-throughput qualitative and quantitative analysis of drug checking samples. The use of online-based m/z cloud library for untargeted compound search improved the ability to identify unknown compounds. Sixty-seven drug checking samples were analyzed using this analytical strategy, allowing the detection of 10 designer drugs and several classical drugs of abuse (mainly cocaine and MDMA) as well as adulterants and contaminants. The results were then compared with routine analyses of the same samples using conventional approaches showing similar performance while removing the use of chromatographic separation thus resulting in a significant reduction of the time required for sample preparation and analysis. This study enlightens the potential of MALDI-HRMS as a high-throughput approach allowing to speed-up up to six times the identification and quantification of substances enabling to catch the fast changes on the drug of abuse market. This strategy could be an interesting alternative analytical approach, allowing better prevention and harm reduction for drug users.

## Introduction

The use of recreational drugs is widespread worldwide, and both the number of users and the diversity of substances are increasing. In Europe, for instance, almost 25% of adults reported at least one illicit drug consumption in their life (Liakoni et al., 2018). In Australia, the situation is more concerning, indeed, in 2016, more than 40% of the population over 14 years old reported a drug use during their lifetimes and more than 15% during the month preceding the survey (Day et al., 2018). Illicit drugs are a global public health issue, since both their acute and chronic consumption have pernicious impacts on the consumer's health (Crowley et al., 2017; Joye et al., 2017; Vearrier, 2019). For instance, it is estimated that almost 10,000 overdose deaths occurred in Europe in 2017 (EMCDDA, 2019).

The risk associated with drug consumption has also increased with the emergence of new psychoactive substances (NPS) or designer drugs leading to a significantly growing number of emergency admissions and fatal overdoses (Miliano et al., 2016). NPS are a rapidly evolving class of substances with various physico-chemical properties and toxicological effects. This class of molecules can be defined as all the substances that are not controlled by the United Nations Single Convention on Narcotic Drugs or the United Nations Convention on Psychotropic substances but might pose public health issues and are often “classical” drugs of abuse derivatives (Khaled et al., 2016). Between 2012 and 2017, more than 400 NPS were monitored for the first time (Elliott et al., 2018). The risk with those substances is that only limited information is available regarding their toxicity for both the drug user and the healthcare professionals (Wood et al., 2016). Various analytical procedures have been published for NPS monitoring in classical and alternative matrices, especially regarding new synthetic opioids (Pichini et al., 2017; Zawilska, 2017; Marchei et al., 2018). For instance, screening procedures targeting a wide range of NPS and designer stimulants as well as studies focusing on designer benzodiazepines have been reported (Adamowicz and Tokarczyk, 2016, 2019; Zawilska and Wojcieszak, 2019). The development of those procedures alongside with the sharing of information through early warning systems is capital to reduce the risk of intoxications and fatalities associated with NPS consumption.

Various drug checking facilities have emerged in the past 20 years. Those facilities allow the drug users to chemically analyze their drugs to check for the presence of adulterants or other non-expected substances without encouraging drug consumption (Sande and Sabic, 2018). Such harm reduction programs provide several advantages such as gaining contact with hard-to-reach target group to provide information and counsel for an increased prevention (Hungerbuehler et al., 2011). The analysis of those substances also provides information regarding the prevalence of drug consumption including NPS arriving on the market and allows the monitoring of a potential altered substance presenting risks. Indeed, evidence also suggests the potential synergistic effect of certain toxic adulterants associated with the illicit drugs leading to overdoses, severe health consequences, and even deaths (Solimini et al., 2017; Singh et al., 2020).

On an analytical point of view, the continuous emergence of new drugs presents an ongoing challenge for clinical and forensic toxicology. Neither the serious toxicity or impairment caused by such substances, nor the analytical methods for their detection and identification is well-established (Peters and Martinez-Ramirez, 2010). To adapt to this constant apparition of new substances and the importance of controlling the substances consumed by drug users, it is necessary to develop analytical tools enabling fast and reliable screening.

Among those analytical approaches, matrix-assisted laser desorption/ionization (MALDI) allows a quick and simple sample preparation. A wide range of use of MALDI technology has been reported for drug monitoring. For instance, MALDI ionization has been used for drug analysis in hair (Vogliardi et al., 2010; Porta et al., 2011; Flinders et al., 2017) and drug mapping in organs or whole-body tissue sections for pharmacodynamic or toxicodynamic studies (Lietz et al., 2013; Sun and Walch, 2013; Patel et al., 2015). Interestingly, an Austrian research group reported the successful use of MALDI combined with high-resolution (HR) MS for the qualitative analysis on drug checking samples focusing on designer drugs (Ostermann et al., 2014). Indeed, the introduction of HRMS analyzers and especially Orbitrap technology increases mass accuracy, allowing the facilitation of identification by reducing the number of possible chemical formulas (Jagerdeo and Schaff, 2016; Joye et al., 2020). Moreover, by improving the mass resolution power, HRMS increases the selectivity, therefore reducing the potential interferences (Chindarkar et al., 2014). Therefore, MALDI-HRMS technology seems to be an interesting alternative to classical GC and LC-MS/MS analyses providing a fast high-throughput complementary approach with a high identification power.

The present study was performed in collaboration with the association “Nuit Blanche” whose goal is to limit the risks associated with drug consumption in the nightlife context of the Swiss Canton of Geneva. This association collects drug checking samples on a weekly basis that are routinely analyzed by GC-MS regarding the screening approach and by LC-MS for the quantitative analyses focusing on 12 substances. Herein, we present a MALDI-HRMS procedure allowing the high-throughput identification of a wide range of drugs of abuse present in drug checking samples based on the online m/z cloud library for untargeted compound search.

The method was applied to 67 real drug checking cases revealing several NPS among the analyzed samples, as well as adulterants. Globally, the method resulted in similar performances than the conventional routine analyses with a significant reduction of the analysis time. In the meantime, the samples were quantified for the most detected drugs of abuse using a single and simple sample preparation. To demonstrate the potential of MALDI quantification in the context of drug checking samples, we further performed a validation according to the FDA (Food and Drug Administration) guidelines. The results of this high-throughput qualitative and quantitative approach were then compared with the classical methodology used in routine showing similar results.

## Methods

### Standard and Reagents

Drug checking samples were provided by the harm reduction association “Nuit Blanche.” LSD, methamphetamine, MDEA, MDMA, amphetamine, mephedrone, and cocaine, as well as their deuterated analogs, were purchased as standards at 1 mg/ml either from Cerilliant or Lipomed. Acetonitrile (ULC-MS 99.99%), methanol (ULC-MS 99.99%), water (ULC-MS), formic acid 99% (ULC-MS), trifluoroacetic acid, as well as the ammonium formate salt were purchased from Biolsolve. Alpha-Cyano-4-hydroxycinnamic acid (CHCA) was purchased from Sigma-Aldrich.

### Sample Preparation

Regarding MALDI experiments, all samples were prepared at 1 mg/ml and 100 μg/ml for qualitative and quantitative analyses, respectively. For qualitative analysis, 10 μl of the sample solution was mixed with 10 μl of matrix solution (CHCA 1 mg/ml, 1:1 ACN: H_2_O + 0.1% TFA). Concerning quantitative analyses, 5 μl of sample solution was first mixed with 5 μl of internal standard (IS) solution. Ten microliters of matrix solution was then added to the mix. The IS solution was prepared containing deuterated LSD, methamphetamine, MDEA, MDMA, amphetamine, mephedrone, and cocaine at 10 μg/ml. Calibration samples were prepared by spiking MeOH at five concentration levels (six considering a blank) ranging from 0.1 to 50 μg/ml with the same undeuterated substances than in the IS solution; 1.5 μl of sample/matrix solution was then spotted on a MALDI stainless steel samples plate for analysis.

An experiment was conducted in order to evaluate the minimal number of acquire spectra to minimize the variability and, therefore, perform accurate quantification. Five samples were prepared using MDMA at 10 μg/ml with a 1:1 drug-to-IS ratio. Five replicates were used to determine intra-spot variability and the relative standard deviation of the signal at 1, 10, 20, 50, 100, and 150 laser shots, respectively.

Routine analyses were performed using two different sample preparations including simple dilutions at 100 μg/ml and 200 ng/ml and a derivation step. Regarding GC-MS screening analyses, a first injection was performed with the diluted samples at 100 μg/ml. For the second injection, 100 μl of the 100 μg/ml sample solution was mixed with 100 μl of anhydric acetic acid and 100 μl of pyridine for derivation. After vortexing, the samples were incubated at 60°C for 30 min. Samples were then evaporated and reconstituted in 100 μl of MeOH. For the quantitative analyses, the dilution at 200 ng/ml was performed in a 50/50 mix of formate buffer 5 mM at pH 3 and MeOH containing IS at a final concentration of 10 ng/ml. Calibration samples were prepared by spiking MeOH at five concentration levels ranging from 1 to 500 ng/ml.

### MALDI Analyses

**Figure 1:**
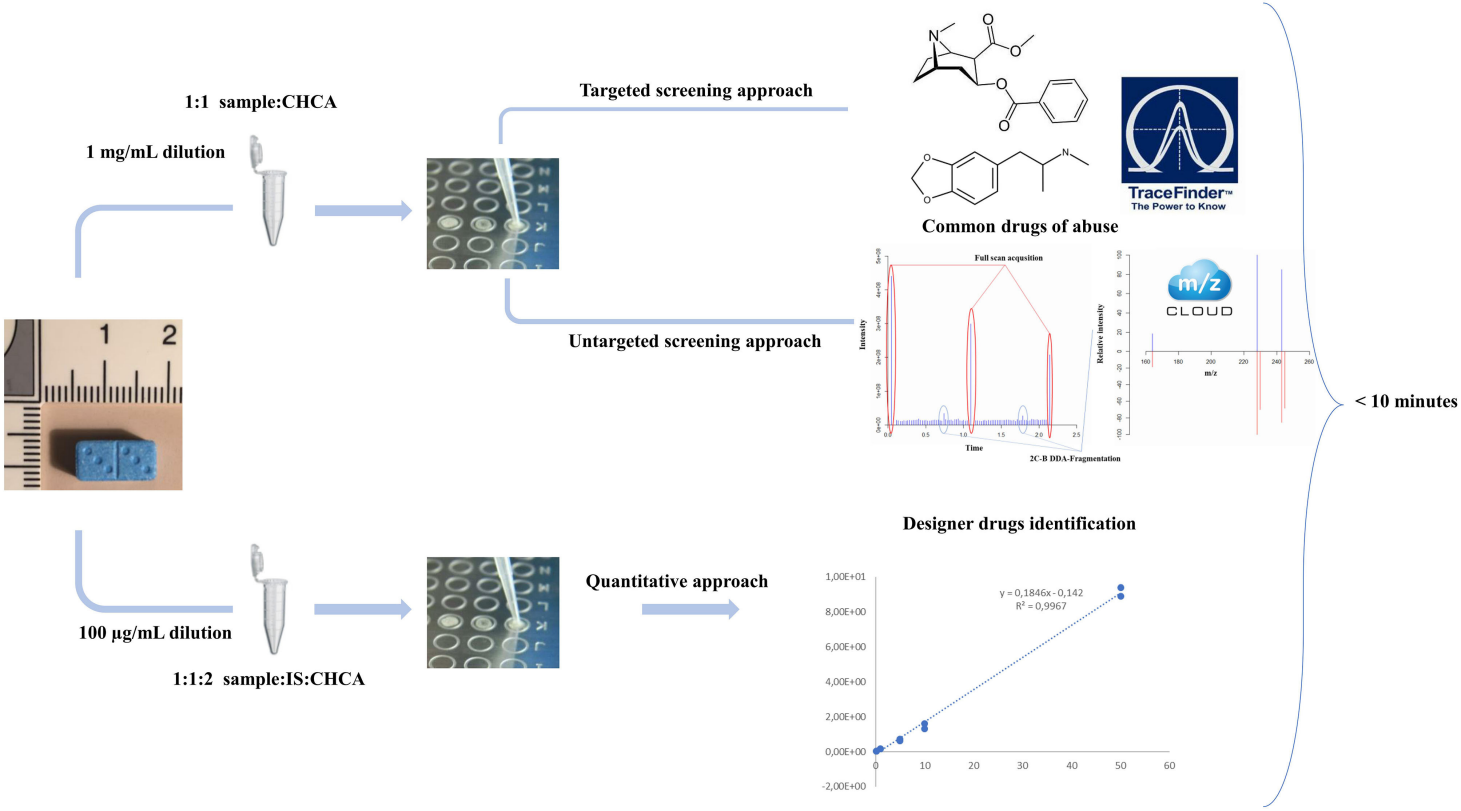
Analytical workflow of the MALDI qual/quant approach including two quick and simple sample preparations.

All samples were analyzed in a three-step process (Figure 1). A first targeted approach targeting the most common drugs of abuse and adulterant was processed, followed by a non-targeted data-dependent acquisition for the detection and identification of other drugs, adulterants, or dangerous contaminants. Then, a full-scan experiment was operated for quantitative analysis.

All MALDI-HRMS experiments were performed using a MALDI-LTQ-Orbitrap XL equipped with a 337-nm N_2_ laser operating at 60 Hz (Thermo Scientific, Bremen, Germany) with a laser beam size of 60 × 50 μm. All analyses were performed with a spectral resolution of 60,000 for full-scan experiments and 17,500 regarding fragmentation. Both the automatic spectral filtering (ASF) and the automatic gain control (AGC) were switched off. MALDI plate motion was set to survey CPS. MALDI laser energy was set to 5 μJ and the number of laser shot was set to five in positive polarity. The scan masses were ranging from 100 to 1,000 m/z.

**Figure 2:**
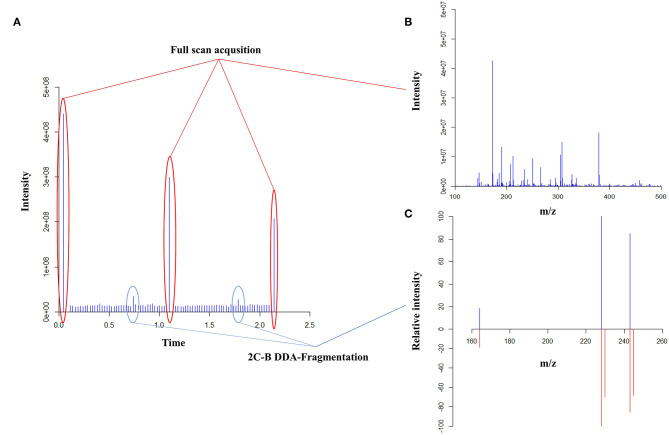
Full scan data-dependent acquisition (DDA) based on the peaks' intensity as an untargeted MALDI screening approach. The total ion current of all acquisition **(A)**, the full-scan spectra **(B)** and a fragmentation spectra comparison of 2C-B **(C)** are represented. In panel C, the top part represents the acquired spectra while the bottom part is the reference spectra.

Regarding qualitative analyses, first, a full-scan data-dependant MS/MS approach was performed using an inclusion list of 41 substances containing the most frequent drugs of abuse and adulterants (Table S1). Fragmentation experiments were performed using higher-energy collisional dissociation (HCD) with normalized collision energy set between 30 and 100 eV depending on the compounds of interest. The second full-scan data-dependent approach was then performed for the detection of other drugs, adulterants or contaminants using a non-targeted approach (Figure 2). Based on the full-scan spectra, the 10 most intense ions were then fragmented using HCD with a normalized energy of 50 eV. A minimum signal threshold was set to 10,000 to avoid fragmenting noise. All fragmented compounds were then sent to an exclusion list already containing the targeted substances for 3 min to avoid fragmenting always the same substances. Quantitative analyses were performed averaging 120 acquired full-scan spectra. The validation criteria used to evaluate the analytical process was based on the directives of the FDA regarding bioanalytical methods and adapted to our specific requirements. The validation was performed over three non-consecutive days (*p* = 3). The trueness and precision were evaluated using a variance analysis-based statistical treatment (ANOVA). Calibration (Cal) was performed in duplicate at five different concentration levels (*k* = 5) (Table 1) while quality controls (QCs) were prepared in quadruplicate at the two lowest and highest concentration levels (*k* = 4). Using the acquired data, trueness, precision, accuracy, and linearity were determined.

**Table 1:** Results for trueness, precision, and linearity (*k* is the number of concentration levels, *n* the number of repetitions by levels, and *p* is the number of non-consecutive days).

**Trueness (%) (*****k*** **=** **4;** ***n*** **=** **4;** ***p*** **=** **3)**				
Calibration level (ng/ml)	100	1,000	10,000	50,000
MDMA	102.7	103.3	109.0	100.7
Cocaine	97.3	103.9	114.4	99.6
**Repeatability/Intermediate precision (RSD%) (*****k*** **=** **4**, ***n*** **=** **4**, ***p*** **=** **3)**				
Calibration level (ng/ml)	100	1,000	10,000	50,000
MDMA	11.6/11.1	13.0/12.3	9.3/10.5	7.0/7.6
Cocaine	12.2/13.7	10.2/11.6	5.9/7.6	4.3/3.9
**Linearity (*****k*** **=** **4**, ***n*** **=** **4**, ***p*** **=** **3)**				
	**Range (ng/ml)**	**Slope**	***R***^**2**^	**Exact mass (m/z)**
MDMA	100–50,000	1.0037	0.9913	194.1176
Cocaine	100–50,000	0.9909	0.9966	304.1543

### Routine Analyses

GC screening analyses were performed using an existing published procedure on a similar instrument (Lefrancois et al., 2016). For the LC-MS/MS quantitative analyses, a Dionex UltiMate 3000 LC-system (Thermo Fischer Scientific, Germany) was used. Gradient elution was performed on a Chromolith Performance RP-C18 (100 × 3 mm) column using a 5 mM formate ammonium buffer at pH 3 (mobile phase A) and acetonitrile (mobile phase B). The gradient and flow rate were programmed as follows: 0–0.2 min hold at 2% B; 0.2–8 min linear increase to 70% B; 8–10 min linear increase to 95% of B; 10–11 min hold at 95% B; 11–14 min hold at 2% B at a constant flow rate of 0.6 ml/min. The injection volume was set to 1 μl. The LC system was coupled with a QTRAP 5500 MS instrument (SCIEX, Netherlands). The mass spectrometer was operated with positive electrospray ionization in multiple reaction monitoring (MRM) mode. The ion spray voltage was set to 5,500 V and the source temperature was 500°C. The gas settings were as follows: Curtain gas: 20 psi, ion source gas 1: 60 psi, ion source gas 2: 40 psi.

### Data Analysis

Information regarding the suspected sample's composition was almost always provided by the drug user. Then, MALDI data were analyzed using Tracefinder (Thermo Scientific) and a database created for this specific drug checking application. Identification was based on the mass over charge ratio, the isotopic pattern, and the library search based on MS/MS spectra comparison. Regarding the samples containing a substance that was not indexed in the library, Xcalibur was used combined with different external library search such as Metlin or m/z cloud. The data were analyzed using MSD Enhanced ChemStation (Agilent Technologies) and compound characterization was performed using mass spectra computerized databases, such as NIST Version 2014 (National Institute of Standards and Technology), Wiley Edition 10, MPW Version 2011 (Maurer, Pfleger, Weber, Drugs, Poisons, Pesticides, Pollutants, and Metabolites), DD Version 2014 (Drug Design and Discovery) and custom databases from the University Institutes of Legal Medicine of the Faculty of Medicine of Geneva (CURML). Quantitative results were treated using Analyst software version 1.6.2.

## Results and Discussion

### MALDI Qualitative Analyses

Consumption of drugs of abuse is a global public health issue. In particular, the emergence of NPS including the classes of phenethylamine and tryptamine emphasizes the risk for the population. Therefore, the development of new fast screening procedures allowing the identification of known and unknown drugs of abuse is a priority to keep up with the ongoing developments on the illicit drug market. The association of highly concentrated analytes with the increased selectivity brought by HRMS technology allows the facilitation of high-throughput MALDI detection and identification of various unknown substances.

With the developed MALDI-HRMS method, several designer drugs were successfully identified including 2,5-dimethoxy-4-methylamphetamine (DOM), 2,5-dimethoxy-4-bromoamphetamine (DOB), 2,5-dimethoxy-4-chloroamphetamine (DOC), 2,5-dimethoxy-4-bromophenethylamine (2C-B) and its position isomer 2-Br-4,5-DMPEA, 2,5-dimethoxy-4-ethylphenethylamine (2C-E), methoxetamine, 4-hydroxy-N-methy-N-ethyltryptamine (4-HO-MET), 6-(2-aminopropyl)benzofuran (6-APB), and clephedrone. Moreover, the MALDI analysis allowed the identification of various adulterants, contaminations, impurities, and synthesis precursors such as safrole, which is used for the synthesis of MDMA.

**Figure 3:**
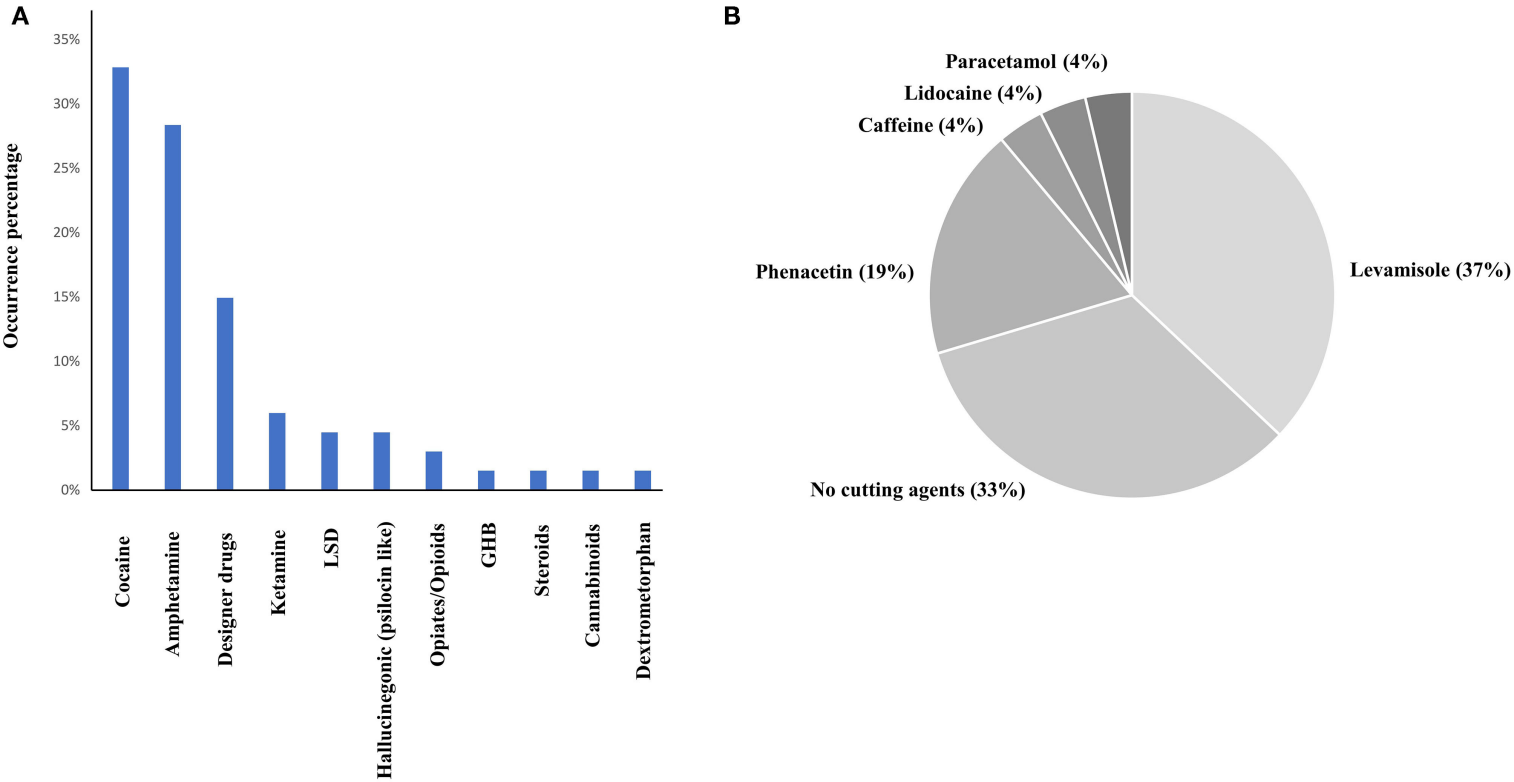
Active principles **(A)** and cocaine cutting agents **(B)** repartition among 67 drug checking samples. Twenty-two cocaine samples were analyzed with an average purity of 71%.

**Figure 4:**
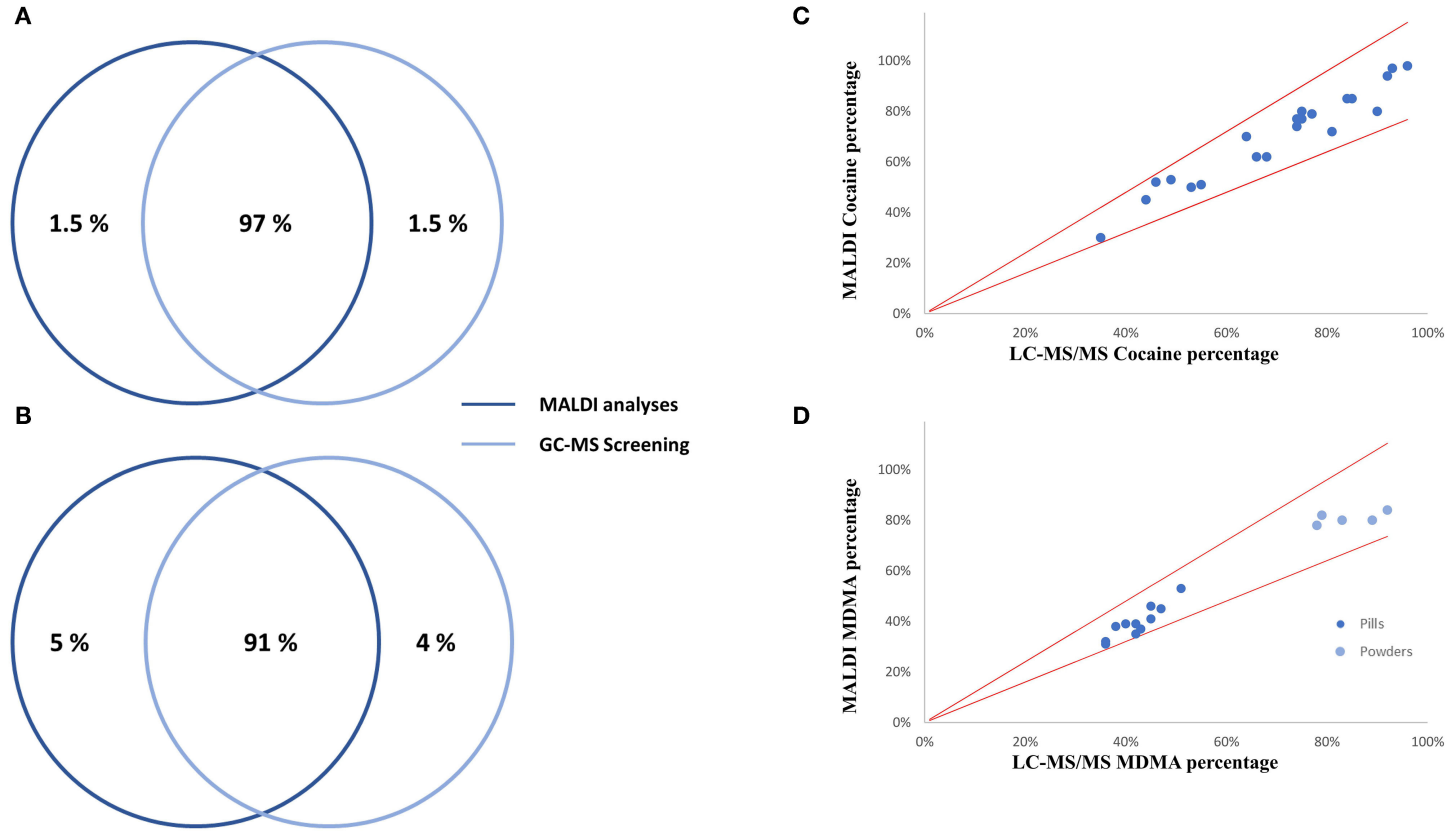
Performance comparison of the developed MALDI qual/quant approach with the routine analyses. Qualitative comparison for actives principles **(A)** and considering all substances **(B)**, as well as quantitative analyses for cocaine **(C)** and MDMA **(D)** are represented.

In total, 67 drug checking samples were analyzed with the developed procedure leading to 101 identifications including 36 different substances (Table S2). Despite the identification of several designer drugs, most samples contained classical drugs of abuse, with 22 samples positive to cocaine and 16 samples positive to MDMA. All the active principles detected using both approaches are represented in Figure 3A. On average, the cocaine purity detected in the 22 samples was high (71%), among them, 10 were containing levamisole, while 9 of them were not containing any cutting agents (Figure 3B). Only classical cocaine cutting agents were identified, yet the identification of such substances is of importance since evidence suggests the concomitant role of certain adulterants and illicit drugs on toxicity (Solimini et al., 2017). The GC-MS screening approach, which is the gold standard for qualitative analyses, showed similar performances to the developed MALDI procedure. Indeed, only one active principle (GHB) was not detected using MALDI-HRMS. Among the cutting agents, metabolites, precursors and alkaloids, one cocaine metabolite (tropacocaine in two samples), and one adulterant (phenacetine) were not detected by the developed approach. On the other hand, one active principle (clephedrone), one cocaine adulterant (levamisole in two samples), one cocaine alkaloid (cinnamoylcocaine), and one MDMA precursor (safrole) were not detected by the GC-MS approach (Figure 3). With a coverage of 98.5% regarding the active principles and 91% considering all the substances (see Figures 4A,B), the main difference between those two approaches was that the use of MALDI analyses allowed the reduction of the time spent for the sample preparation with the removal of the derivation process and the analysis by a six times factor reaching a total analysis times of around 10 min per sample.

### MALDI Quantitative Analyses

**Figure 5:**
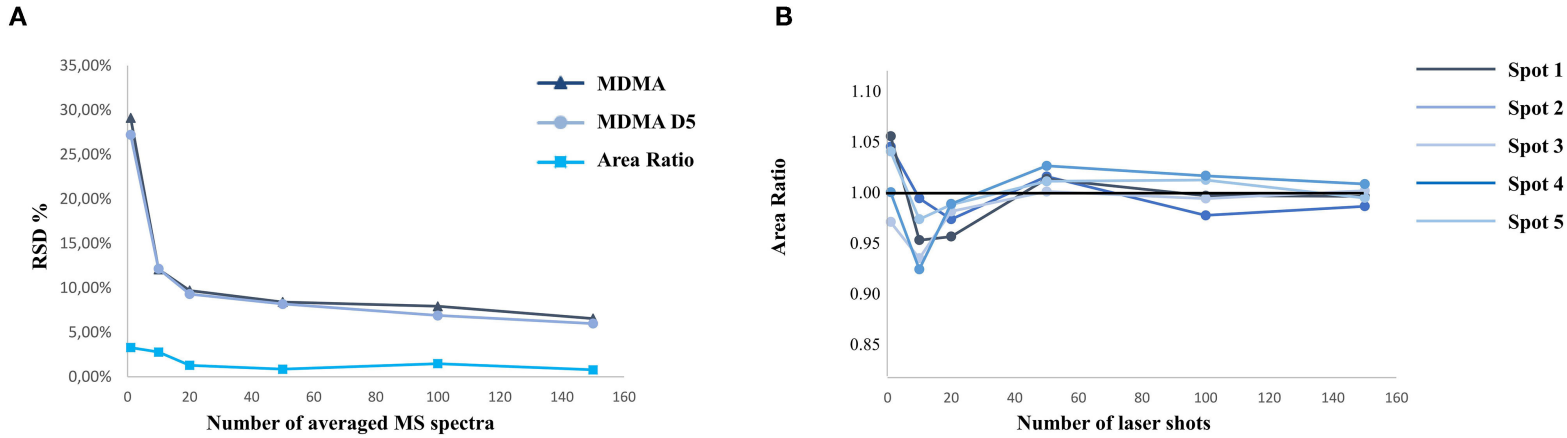
Relative standard deviation (%) associated with MALDI analysis as a function of the number of averaged acquisition points **(A)**. Intra-spot variability and accuracy depending on the number of laser shot averaged per acquisition **(B)**.

MALDI analyses are often associated with a relatively important variability regarding signal intensity. Therefore, MALDI quantification requires specific development to ensure good results in terms of accuracy, precision, and repeatability (Porta et al., 2015). One solution to limit this specific signal variability issue is to normalize the intensity obtained by averaging enough acquired pixels. One of the challenges is to find the right compromise between the time of acquisition associated with the number of averaged spectra and a good accuracy and precision. During this study, tests were performed to optimize the acquisition time with a limited variability. As demonstrated in the literature, our results enlightened the need for isotopically labeled IS and acquisition of a sufficient number of spectra to decrease relative standard deviation below 10% (Figure 5) (Ostermann et al., 2014; Porta et al., 2015). As demonstrated in Figure 5, the acquisition of more than 100 averaged spectra was thus necessary to obtain good repeatability and accuracy.

**Figure 6:**
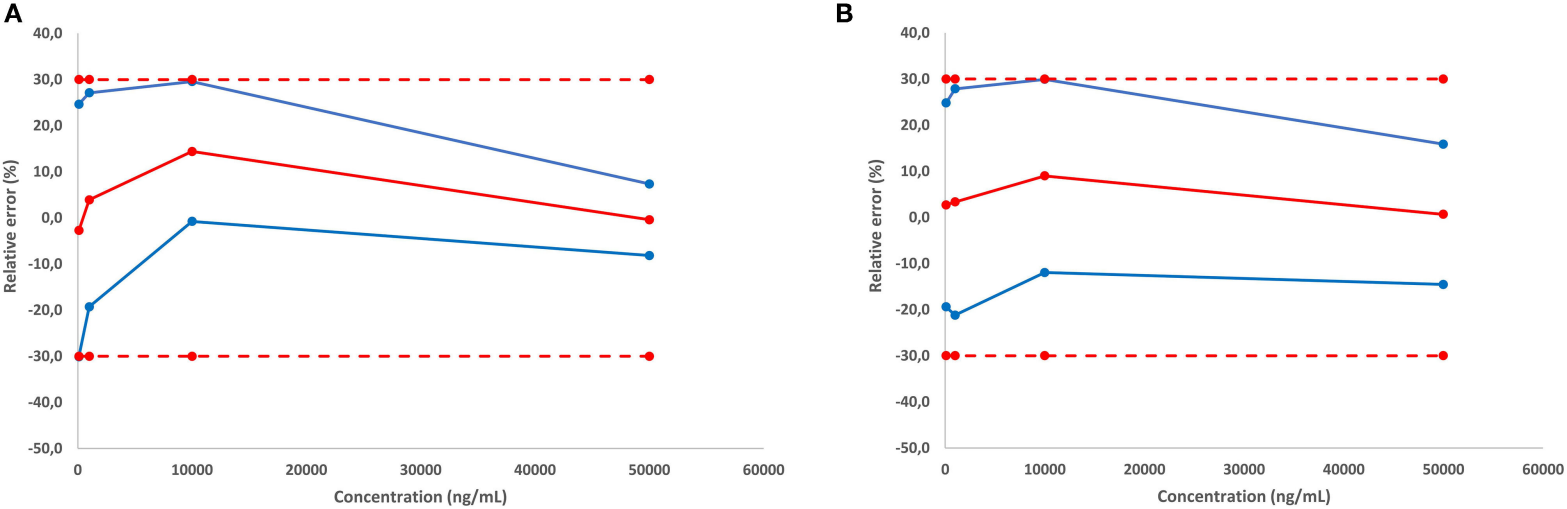
Accuracy profile for cocaine **(A)**, and MDMA **(B)**.

The validation of the quantitative approach was performed for cocaine and MDMA by analyzing independent QC samples in quadruplicates at four different calibration levels over three non-consecutive days for the determination of trueness, precision, and linearity. Accuracy represents the total error and can be divided into two parameters including trueness (bias or systematic error) and precision (the standard deviation or random errors). Trueness is calculated using the percentage difference between the experimental and the expected values. In the present study, trueness was ranging from −2.7 to 14.4% (Table 1). Precision was divided into repeatability and the inter-day variability (intermediate precision). Repeatability represents the variability under similar conditions performed by the same operator while intermediate precision is the variability associated with the use of the same samples on different days with different reagents. Repeatability and intermediate precision were measured between 3.9 and 13.7% (Table 1). Accuracy profiles are visual representations of the uncertainty measurement combining the trueness and precision (Figure 6). Precision is represented by the calculated confidence limit at 95% for each concentration limit. Accuracy profiles also include the representation of acceptance limits of ±30% suggested for method validation. Linearity is defined as the method capacity to provide a result proportional to the real sample concentration. Its determination requires linear regression model based on the least square method applied on the fit of the obtained concentrations as a function of the expected concentrations. Slope values were calculated at 1.0037 and 0.9909 with coefficients of determination of 0.9913 and 0.9966 for MDMA and cocaine, respectively.

Overall, MALDI quantitative results showed a good correlation with the routine LC-MS/MS MRM experiments. Indeed, all MALDI results were in agreement with the routine analysis with a ±20% tolerance window. The correlation between the two analytical strategies is represented in Figure 4 for cocaine and MDMA, being the two most detected substances. Interestingly, among all MDMA-positive cases, two groups can be distinguished. Indeed, all the samples in the form of pills had an MDMA percentage between 31 and 52%, while this percentage was measured between 78 and 84% regarding powders (Figure 4D). MALDI quantitation analyses were performed in around 3 min per sample while one LC injection lasted for 14 min. With the HRMS quantification being based on full-scan spectra, the addition of new substances of interest can easily be performed without the need of any preliminary developments such as infusion processes.

## Conclusion

In this study, we enlighten the potential of MALDI-HRMS as a high-throughput analytical strategy in forensic and clinical toxicology. This technology can bring interesting applications despite the absence of chromatographic separation, which may be detrimental for the analysis of low-concentration analytes in complex matrices. Nevertheless, considering drug checking analysis where the analytes can be concentrated at will, MALDI-HRMS allows one to significantly speed up the detection, identification, and quantification of various drugs of abuse. With the development of bioinformatic tools and online shared libraries such as m/z cloud, the method can easily be adapted for any new substance appearing on the market being in agreement with the challenges brought by the continuous emergence of NPS.

The developed approach showed similar qualitative and quantitative results for drug checking compared to those obtained from both LC-MS and GC-MS while reducing by six times the analytical procedure. The development of such rapid drug checking strategies would enable faster monitoring of changes in the drug market, providing an improved tool for prevention and harm reduction for drug users.

## Data Availability Statement

The raw data supporting the conclusions of this article will be made available by the authors, without undue reservation.

## Author Contributions

TJ developed the analytical method did the samples analyses and wrote the manuscript. CW and MA participated in the strategic choices toward the study and its design and critically revised the manuscript. RM and SL provided the samples and critically revised the manuscript. AT was the main contributor to the study design, supervised the project, and critically revised the manuscript. All authors contributed to the article and approved the submitted version.

## Conflict of Interest

The authors declare that the research was conducted in the absence of any commercial or financial relationships that could be construed as a potential conflict of interest.
