# Supplementary material for: High-Throughput Qualitative and Quantitative Drug Checking by MALDI HRMS
Source: Front Chem. 2020 Aug 25;8:695. doi: 10.3389/fchem.2020.00695 (PMC7477897; doi:10.3389/fchem.2020.00695)
Supplement: Supplementary file 1 [file Table_1.DOCX]

**Supplemental Table 1 :**

| Exact Mass [M+H]+ | Compound |
| --- | --- |
| 136.1120 | Amphetamine |
| 150.1277 | Methamphetamine |
| 152.0706 | Paracetamol |
| 177.1386 | Benzylpiperazine |
| 178.1226 | Mephedrone |
| 180.1019 | Phenacetin |
| 192.1383 | 4-MEC |
| 194.1176 | MDMA |
| 195.0877 | Caffeine |
| 205.0794 | Levamisole |
| 208.0968 | Methylone |
| 208.1332 | MDEA/MBDB |
| 210.1489 | DOM/2C-E |
| 234.1489 | Methylphenidate |
| 235.1805 | Lidocaine |
| 238.0993 | Ketamine |
| 246.1489 | Tropacocaine |
| 248.1645 | Ethylphenidate |
| 260.0281 | 2C-B |
| 264.1958 | Tramadol |
| 274.0437 | DOB |
| 271.0627 | Nordiazepam |
| 276.1594 | MDPV |
| 278.1895 | Amitriptyline |
| 286.1438 | Morphine |
| 287.0808 | Oxazepam |
| 290.1594 | Benzoylecgonine |
| 300.1594 | Codeine |
| 304.1543 | Cocaine |
| 308.1757 | Zolpidem |
| 310.2165 | Methadone |
| 324.2070 | LSD |
| 325.1704 | Citalopram |
| 326.0855 | Midazolam |
| 328.1543 | 6-MAM |
| 330.1700 | 25-NBOMe/Cinnamyolcocaine |
| 337.2274 | Fentanyl |
| 340.1543 | Papaverine |
| 353.0786 | Griseofulvin |
| 370.1649 | Heroine |
| 414.1547 | Noscapine |
