# Supplementary material for: High-Throughput Qualitative and Quantitative Drug Checking by MALDI HRMS
Source: Front Chem. 2020 Aug 25;8:695. doi: 10.3389/fchem.2020.00695 (PMC7477897; doi:10.3389/fchem.2020.00695)
Supplement: Supplementary file 2 [file Table_2.DOCX]

**Supplemental Table 2 :**

| Cases | GC-MS Screening | MALDI HRMS Screening | Announced active principle |
| --- | --- | --- | --- |
| 1 | DOM | DOM | DOM |
| 2 | MDMA | MDMA | MDMA |
| 3 | Cocaine | Cocaine | Cocaine |
|  | Tropacocaine |  |  |
| 4 | Cocaine | Cocaine | Cocaine |
| 5 | MDMA | MDMA | MDMA |
| 6 | MDMA | MDMA | MDMA |
| 7 | Cocaine | Cocaine | Cocaine |
| 8 | MDMA | MDMA | MDMA |
| 9 | Ketamine | Ketamine | Ketamine |
| 10 | Methamphetamine | Methamphetamine | Methamphetamine |
| 11 | 2C-B | 2C-B | 2C-B |
| 12 | Cocaine | Cocaine | Cocaine |
|  | Caffeine | Caffeine |  |
|  | Cinnamoylcocaine | Cinnamoylcocaine |  |
|  |  | Levamisole |  |
| 13 | Cocaine | Cocaine | Cocaine |
| 14 | LSD | LSD | LSD |
| 15 | MDMA | MDMA | MDMA |
|  |  | Safrole |  |
| 16 | LSD | LSD | LSD |
| 17 | Cocaine | Cocaine | Cocaine |
| 18 | Cocaine | Cocaine | Cocaine |
| 19 | Cocaine | Cocaine | Cocaine |
| 20 | Ketamine | Ketamine | Ketamine |
| 21 | Cocaine | Cocaine | Cocaine |
|  | Lidocaine | Lidocaine |  |
|  | Phenacetin | Phenacetin |  |
|  | Levamisole | Levamisole |  |
| 22 | MDMA | MDMA | MDMA |
| 23 | CBD | CBD | CBD |
| 24 | Kétamine | Kétamine | Kétamine |
| 25 | Cocaine | Cocaine | Cocaine |
|  | Tropacocaine | Tropacocaine |  |
|  | Cinnamoylcocaine | Cinnamoylcocaine |  |
|  | Lévamisole | Lévamisole |  |
|  | Phénacétine | Phénacétine |  |
| 26 | Cocaine | Cocaine | Cocaine |
|  | Tropacocaine | Tropacocaine |  |
|  | Phénacétine | Phénacétine |  |
| 27 | Cocaine | Cocaine | Cocaine |
|  | Tropacocaine |  |  |
|  | Phénacétine |  |  |
|  |  | Cinnamoylcocaine |  |
|  |  | Levamisole |  |
| 28 |  |  | Ethanol |
|  | Methadone | Methadone |  |
| 29 | GHB | ND | GHB |
| 30 | Amphetamine | Amphetamine | Amphetamine |
| 31 | MDMA | MDMA | MDMA |
| 32 | MDMA | MDMA | MDMA |
| 33 | MDMA | MDMA | MDMA |
| 34 | Cocaine | Cocaine | Cocaine |
| 35 | MDMA | MDMA | MDMA |
| 36 | Amphetamine | Amphetamine | Amphetamine |
| 37 | 2-Br-4,5-DMPEA | 2-Br-4,5-DMPEA | 2C-B |
| 38 | MDMA | MDMA | MDMA |
| 39 | Stanozolol | Stanozolol | Metenolone |
| 40 | LSD | LSD | LSD |
| 41 | MDMA | MDMA | MDMA |
| 42 | Cocaine | Cocaine | Cocaine |
|  | Levamisole | Levamisole |  |
| 43 | Cocaine | Cocaine | Cocaine |
|  | Cinnamoylcocaine | Cinnamoylcocaine |  |
| 44 | Cocaine | Cocaine | Cocaine |
|  | Phenacetin | Phenacetin |  |
| 45 | Cocaine | Cocaine | Cocaine |
|  | Levamisole | Levamisole |  |
|  | Paracetamol | Paracetamol |  |
| 46 | Methoxetamine | Methoxetamine | Methoxetamine |
| 47 | 4-HO-MET | 4-HO-MET | 4-HO-MET |
| 48 | Cocaine | Cocaine | Cocaine |
| 49 | Cocaine | Cocaine | Cocaine |
|  | Levamisole | Levamisole |  |
| 50 | ND | Clephedrone | Mephedrone |
| 51 | Cocaine | Cocaine | Cocaine |
|  | Levamisole | Levamisole |  |
| 52 | Cocaine | Cocaine | Cocaine |
|  | Levamisole | Levamisole |  |
|  | Tropacocaine | Tropacocaine |  |
| 53 | MDMA | MDMA | MDMA |
| 54 | MDMA | MDMA | MDMA |
| 55 | Cocaine | Cocaine | Cocaine |
|  | Tropacocaine | Tropacocaine |  |
|  | Levamisole | Levamisole |  |
| 56 | Psilocine | Psilocine | Unknown |
| 57 | 2C-E | 2C-E | 2C-E |
| 58 | DOC | DOC | DOC |
| 59 | MDMA | MDMA | MDMA |
| 60 | DOB | DOB | DOB |
| 61 | 4-ACO-DMT | 4-ACO-DMT | 4-ACO-DMT |
| 62 | DMT | DMT | DMT |
| 63 | MDMA | MDMA | MDMA |
| 64 | Ketamine | Ketamine | Kétamine |
| 65 | 6-APB | 6-APB | 6-APB |
| 66 | Heroine | Heroine | Unknown |
|  | Caffeine | Caffeine |  |
|  | Paracetamol | Paracetamol |  |
|  | Codeine | Codeine |  |
|  | Noscapine | Noscapine |  |
|  | Papaverine | Papaverine |  |
| 67 | DXM | DXM | DXM |
